# In Memoriam—Dr. J. Heber Smith

**Published:** 1898-12

**Authors:** 


### IN MEMORIAM—DR. J. HEBER SMITH.

J. Heber Smith, M. D., of Boston, died in this city of heart disease Sunday morning, October 23d. He was born in Bucksport, Me., December 5th, 1842, and was the son of Rev. Joseph Smith, a somewhat widely known Methodist clergyman of New England.

In early life Dr. Smith was prevented by ill-health from completing a classical course at Harvard College, for which he was prepared. His health afterwards improved, and he entered with enthusiasm upon the study of medicine. He was graduated at the Hahnemann Medical College of Philadelphia in March 1864, as the valedictorian ,of his class. Almost immediately he entered upon a successful practice in Melrose which continued till 1882, when he removed to Boston, where he had been often previously called in consultation, and where he had since continued in practice.

In 1873, on the foundation of Boston University School of Medicine, Dr. Smith became one of its original members as Professor of Materia Medica, a position he filled with great ability to the present time. Since 1878 he has been one of its executive committee and its secretary. As a professor for more than twenty-five years he seldom failed to promptly meet its requirements. His lectures were carefully prepared and filled with important information. His manner was attractive and impressive, and not one of the many hundreds who have been his pupils but appreciated the valuable instruction received from him.

As a physician he was devoted to the interests of his patients, and he will long be enshrined in their memory. For more than thirty years he had been an active member of the American Institute of Homœopathy, the Massachusetts Homœopathic Medical Society, of which he was president in 1884, and of the Boston society, to all of which he contributed valuable papers. He had also been a member of many other societies and associations.

He leaves a widow and two children—Mrs. Horace G. Lobenstine, a married daughter, who resides in Detroit, and a son, Conrad Smith, who has nearly completed his medical education.

The funeral will be at his former home to-morrow at three o'clock.—*Boston Transcript*, October 24th, 1898.

---

On Tuesday, October 25th, at twelve o'clock, the Faculty of Boston University School of Medicine held a meeting at the school building to take action on the death of Professor J. Heber Smith.

The meeting was presided over by the Dean, Dr. I. T. Talbot. Dr. H. C. Clapp read an article which had been prepared for one of the daily papers, presenting a brief biographical sketch of Dr. Smith. Members of the Faculty then testified, each in a few words, to their appreciation of the many qualities possessed by their late colleague. They spoke of his great courage, of his striking faithfulness to the School, of the clearness of his teaching, his constant cordiality and cheerfulness, his magnetic personality, and many other noble and lovable traits which had won for him the regard of Faculty and students alike. Dr. Richardson, when called upon for remarks, read some verses by the late Sherman Hoar, beginning:

"Give unto Thy servant rest,"

which seemed to be peculiarly appropriate to the occasion.

Of the thirty-two members who represented the Faculty at the meeting the following took part in the exercises:

Drs. E. P. Colby, .F B. Percy, J. W. Hayward, Alonzo Boothby, J. A. Rockwell, Conrad Wesselhœft, Horace Packard, F. P. Batchelder, Prof. E. E. Calder, Drs. W. S. Smith, Geo. S. Adams, J. E. Briggs, F. E. Allard, Marion Coon, Sarah S. Windsor, F. A. Davis, W. T. Hopkins, Martha Mann, C. H. Thomas, A. H. Powers, J. S. Shaw, Walter Wesselhœft, F. C. Richardson, H. C. Clapp, and W. T. Talbot.

Among those coming from a distance were Dr. Adams, of Westborough, and Prof. Calder, of Providence. The following resolutions, which had been prepared by a committee previously appointed, were read by Dr. Sutherland and unanimously adopted by a rising vote:

J. Heber Smith, physician, medical teacher, friend, having been called by the dispensation of the Eternal Wisdom from his earthly labors, his surviving colleagues on the Faculty of Boston University School of Medicine mourn his death, honor his memory, and hereby testify to their deep appreciation of his quarter of a century's unremitting, steadfast, and faithful labors in behalf of the school. In class-room, in business meeting, in social gathering, his clear and efficient teaching, his words of counsel, and his genial presence will be sadly missed. His strong individuality, his unfailing cheerfulness, constant good humor, and pungent wit, united with his scholarly attainments, made him a convincing personality. His patient and uncomplaining submission to life-long infirmity, his sympathetic and keen appreciation of the sufferings of others, his energy and forgetfulness of self in administering to the necessities of others will linger as an example to be imitated by all whose good fortune it was to know him.

To his family and relatives we extend our sincerest sympathy for a bereavement which is an affliction shared by all who were numbered with his friends.

J. P. SUTHERLAND,
H. C. CLAPP,
J. W. HAYWARD,
*Committee.*

The following members of the Faculty acted as honorary pall-bearers: Drs. Talbot, Sutherland, Conrad Wesselhœft, and H. C. Clapp.

